# Supplementary material for: A fault-intrusion-tolerant system and deadline-aware algorithm for scheduling scientific workflow in the cloud
Source: PeerJ Comput Sci. 2021 Nov 2;7:e747. doi: 10.7717/peerj-cs.747 (PMC8576554; doi:10.7717/peerj-cs.747)
Supplement: Supplemental Information 6 [file peerj-cs-07-747-s006.zip › Myproject1.1/docs/org/cloudbus/cloudsim/power/class-use/PowerVmAllocationPolicyMigrationAbstract.html]

Uses of Class org.cloudbus.cloudsim.power.PowerVmAllocationPolicyMigrationAbstract


JavaScript is disabled on your browser.


- Overview
- Package
- Class
- Use
- Tree
- Deprecated
- Index
- Help

- Prev
- Next

- Frames
- No Frames

- All Classes

## Uses of Class org.cloudbus.cloudsim.power.PowerVmAllocationPolicyMigrationAbstract

- Packages that use PowerVmAllocationPolicyMigrationAbstract

  | Package | Description |
  | --- | --- |
  |  |  |
  | --- | --- |
  | org.cloudbus.cloudsim.examples.power |  |
  | org.cloudbus.cloudsim.power |  |
- - ### Uses of PowerVmAllocationPolicyMigrationAbstract in org.cloudbus.cloudsim.examples.power

    Methods in org.cloudbus.cloudsim.examples.power with parameters of type PowerVmAllocationPolicyMigrationAbstract

    | Modifier and Type | Method and Description |
    | --- | --- |
    |  |  |
    | --- | --- |
    | `static void` | Helper.`printMetricHistory(java.util.List<? extends Host> hosts, PowerVmAllocationPolicyMigrationAbstract vmAllocationPolicy)` Prints the metric history. |
    | `static void` | Helper.`writeMetricHistory(java.util.List<? extends Host> hosts, PowerVmAllocationPolicyMigrationAbstract vmAllocationPolicy, java.lang.String outputPath)` Write metric history. |
  - ### Uses of PowerVmAllocationPolicyMigrationAbstract in org.cloudbus.cloudsim.power

    Subclasses of PowerVmAllocationPolicyMigrationAbstract in org.cloudbus.cloudsim.power

    | Modifier and Type | Class and Description |
    | --- | --- |
    |  |  |
    | --- | --- |
    | `class` | `PowerVmAllocationPolicyMigrationInterQuartileRange` The Inter Quartile Range (IQR) VM allocation policy. |
    | `class` | `PowerVmAllocationPolicyMigrationLocalRegression` The Local Regression (LR) VM allocation policy. |
    | `class` | `PowerVmAllocationPolicyMigrationLocalRegressionRobust` The Local Regression Robust (LRR) VM allocation policy. |
    | `class` | `PowerVmAllocationPolicyMigrationMedianAbsoluteDeviation` The Median Absolute Deviation (MAD) VM allocation policy. |
    | `class` | `PowerVmAllocationPolicyMigrationStaticThreshold` The Static Threshold (THR) VM allocation policy. |

    Methods in org.cloudbus.cloudsim.power that return PowerVmAllocationPolicyMigrationAbstract

    | Modifier and Type | Method and Description |
    | --- | --- |
    |  |  |
    | --- | --- |
    | `PowerVmAllocationPolicyMigrationAbstract` | PowerVmAllocationPolicyMigrationInterQuartileRange.`getFallbackVmAllocationPolicy()` Gets the fallback vm allocation policy. |
    | `PowerVmAllocationPolicyMigrationAbstract` | PowerVmAllocationPolicyMigrationLocalRegression.`getFallbackVmAllocationPolicy()` Gets the fallback vm allocation policy. |
    | `PowerVmAllocationPolicyMigrationAbstract` | PowerVmAllocationPolicyMigrationMedianAbsoluteDeviation.`getFallbackVmAllocationPolicy()` Gets the fallback vm allocation policy. |

    Methods in org.cloudbus.cloudsim.power with parameters of type PowerVmAllocationPolicyMigrationAbstract

    | Modifier and Type | Method and Description |
    | --- | --- |
    |  |  |
    | --- | --- |
    | `void` | PowerVmAllocationPolicyMigrationInterQuartileRange.`setFallbackVmAllocationPolicy(PowerVmAllocationPolicyMigrationAbstract fallbackVmAllocationPolicy)` Sets the fallback vm allocation policy. |
    | `void` | PowerVmAllocationPolicyMigrationLocalRegression.`setFallbackVmAllocationPolicy(PowerVmAllocationPolicyMigrationAbstract fallbackVmAllocationPolicy)` Sets the fallback vm allocation policy. |
    | `void` | PowerVmAllocationPolicyMigrationMedianAbsoluteDeviation.`setFallbackVmAllocationPolicy(PowerVmAllocationPolicyMigrationAbstract fallbackVmAllocationPolicy)` Sets the fallback vm allocation policy. |

    Constructors in org.cloudbus.cloudsim.power with parameters of type PowerVmAllocationPolicyMigrationAbstract

    Constructor and Description

    `PowerVmAllocationPolicyMigrationInterQuartileRange(java.util.List<? extends Host> hostList,
    PowerVmSelectionPolicy vmSelectionPolicy,
    double safetyParameter,
    PowerVmAllocationPolicyMigrationAbstract fallbackVmAllocationPolicy)`

    Instantiates a new power vm allocation policy migration mad.

    `PowerVmAllocationPolicyMigrationInterQuartileRange(java.util.List<? extends Host> hostList,
    PowerVmSelectionPolicy vmSelectionPolicy,
    double safetyParameter,
    PowerVmAllocationPolicyMigrationAbstract fallbackVmAllocationPolicy,
    double utilizationThreshold)`

    Instantiates a new power vm allocation policy migration mad.

    `PowerVmAllocationPolicyMigrationLocalRegression(java.util.List<? extends Host> hostList,
    PowerVmSelectionPolicy vmSelectionPolicy,
    double safetyParameter,
    double schedulingInterval,
    PowerVmAllocationPolicyMigrationAbstract fallbackVmAllocationPolicy)`

    Instantiates a new power vm allocation policy migration local regression.

    `PowerVmAllocationPolicyMigrationLocalRegression(java.util.List<? extends Host> hostList,
    PowerVmSelectionPolicy vmSelectionPolicy,
    double safetyParameter,
    double schedulingInterval,
    PowerVmAllocationPolicyMigrationAbstract fallbackVmAllocationPolicy,
    double utilizationThreshold)`

    Instantiates a new power vm allocation policy migration local regression.

    `PowerVmAllocationPolicyMigrationLocalRegressionRobust(java.util.List<? extends Host> hostList,
    PowerVmSelectionPolicy vmSelectionPolicy,
    double safetyParameter,
    double schedulingInterval,
    PowerVmAllocationPolicyMigrationAbstract fallbackVmAllocationPolicy)`

    Instantiates a new power vm allocation policy migration local regression.

    `PowerVmAllocationPolicyMigrationLocalRegressionRobust(java.util.List<? extends Host> hostList,
    PowerVmSelectionPolicy vmSelectionPolicy,
    double safetyParameter,
    double schedulingInterval,
    PowerVmAllocationPolicyMigrationAbstract fallbackVmAllocationPolicy,
    double utilizationThreshold)`

    Instantiates a new power vm allocation policy migration local regression.

    `PowerVmAllocationPolicyMigrationMedianAbsoluteDeviation(java.util.List<? extends Host> hostList,
    PowerVmSelectionPolicy vmSelectionPolicy,
    double safetyParameter,
    PowerVmAllocationPolicyMigrationAbstract fallbackVmAllocationPolicy)`

    Instantiates a new power vm allocation policy migration mad.

    `PowerVmAllocationPolicyMigrationMedianAbsoluteDeviation(java.util.List<? extends Host> hostList,
    PowerVmSelectionPolicy vmSelectionPolicy,
    double safetyParameter,
    PowerVmAllocationPolicyMigrationAbstract fallbackVmAllocationPolicy,
    double utilizationThreshold)`

    Instantiates a new power vm allocation policy migration mad.

- Overview
- Package
- Class
- Use
- Tree
- Deprecated
- Index
- Help

- Prev
- Next

- Frames
- No Frames

- All Classes
